# Knowledge and Practice on Prevention of Respiratory Health Problems among Traffic Police in Kathmandu, Nepal

**DOI:** 10.1155/2015/716257

**Published:** 2015-08-13

**Authors:** Ambika Aryal Bhandari, Roshani Gautam, Shiva Bhandari

**Affiliations:** ^1^Maharajgunj Nursing Campus, Institute of Medicine, Tribhuvan University, Maharajgunj, Kathmandu 44616, Nepal; ^2^Central Department of Microbiology, Tribhuvan University, Kirtipur, Kathmandu 44613, Nepal; ^3^Kantipur College of Medical Science, Tribhuvan University, Sitapaila, Kathmandu 44600, Nepal

## Abstract

*Introduction*. Traffic police in Kathmandu are continuously exposed to air pollution and are at an increased health risk. This study aimed to assess the knowledge and practice regarding prevention of respiratory problems among traffic police in Kathmandu. *Methods*. A descriptive exploratory study was conducted among the traffic police (*n* = 83) working in six areas of the Kathmandu Metropolis from July to August 2013. Self-administered questionnaires were distributed to all the participants. *Results*. The mean age (±SD) of the respondents was 28.8 ± 4.3 years. More than half of the respondents had 6–10 years of work experience, the mean (±SD) years of experience being 7.9 (±3.6). The level of knowledge regarding the prevention of respiratory problems was better than the level of practice among the respondents. Education of the participants did not affect the level of practice of the respondents while there was association between working experience and level of practice (*p* = 0.04). *Conclusion*. Since the preventive practice is poor, the government should come up with plans such as distribution of antipollution masks to improve the level of practice among traffic police to prevent respiratory problems.

## 1. Introduction

Occupational health risks and hazards due to polluted environment have become a serious public health concern where there is unplanned urbanization. Pollution due to road traffic is a serious health hazard and thus the persons like traffic police who are continuously exposed may be at an increased risk [1, 2]. The traffic police have to undergo physical strain in an environment polluted by fumes, exhaust of vehicles, use of blowing horns, emission from nearby brick factories, and blow of dust in the air by a speeding vehicle.

The traffic situation in Kathmandu Metropolis is dominated by an enormous increase of motorized vehicles. Altogether 688,026 vehicles have been added to the Bagmati zone from 1990 to 2013 and most of them are being added to the already congested roads of the Kathmandu Valley [3]. Of the total vehicles, only 2.7% of vehicles are public while 92.1% of vehicles are privately owned [4]. According to a report from Clean Air Network Nepal, motorcycles dominate the vehicles composition by 73.2% and Car/Jeep/Van by 18.5% [5]. A study shows that vehicle flow in Kathmandu is Two-Wheelers (motorcycles) (77.5%), Taxi (8.2%), Car/Jeep/Van (7.6%), Truck/Mini Truck (1.9%), Bus/Minibus (1.7%), and Microbus (1.6%) [6].

Air pollution from vehicles is the complex function of fuel characteristics, extents of combustion, reactions with other gases, and atmospheric condition. The major fuels used in vehicles of Nepal are diesel and petrol. However, adulteration (mainly with kerosene) in such fuels can increase ambient air pollution. A study in Nepal showed that existing diesel (commercially dispensed at automotive fuel pump stations) indicated presence of 30–50% kerosene in diesel [7].

In Nepal, air quality crisis in cities is mainly due to increased vehicular emission, rampant construction works, and unmanaged factories [8, 9]. The World Health Organization (WHO) has found Kathmandu as one of the most polluted cities in Asia with regard to PM_10_ (particulate matter) and PM_2.5_ level in ambient air [10]. Adverse health effects due to air pollution range anywhere from minor irritation of eyes and the upper respiratory system to chronic respiratory disease, heart disease, lung cancer, and death [11]. The particles emitted from the vehicular exhaust of more than 10-micron size are held in upper respiratory tract and particles less than 10-micron size accumulate in the lung and produce respiratory abnormalities [12].

According to the WHO, outdoor air pollution caused about 3.7 million premature deaths worldwide in 2012, 88% of those occurring in low- and middle-income countries [11]. A study conducted in 2006 in Nepal revealed a fact that over 1,900 premature deaths occur per year in Kathmandu Valley due to air pollution [13]. According to the Metropolitan Traffic Police Office in Singha Durbar, as many as 50 traffic police personnel fall ill daily due to hazardous dust on the recently demolished but yet-to-be-reconstructed roads (unpublished data). Similarly, a study showed that the negative effect of air pollution on health of dwellers in Kathmandu Valley is extreme [14]. Therefore, this study aimed to assess the knowledge regarding respiratory problems among traffic police in Kathmandu Valley and common preventive measures adopted by them to prevent respiratory problems.

## 2. Methods

### 2.1. Study Design and Setting

**Figure 1:**
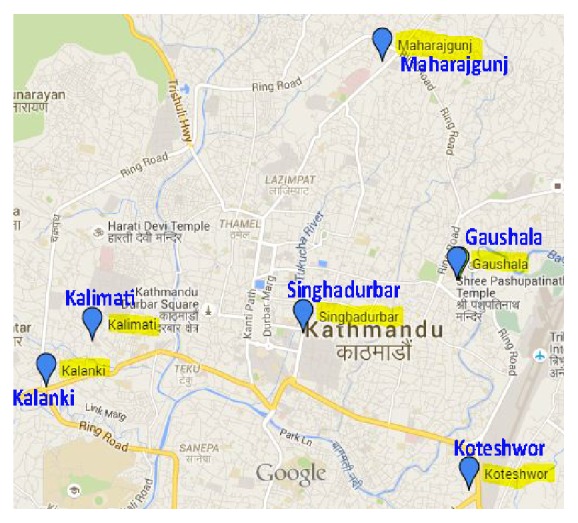
Map of study areas in Kathmandu Metropolis, Nepal.

A descriptive exploratory research was conducted among the traffic police working in six areas of Kathmandu city from July to August 2013. The selected areas were Singha Durbar, Maharajgunj, Kalanki, Koteshwor, Gaushala, and Kalimati. These areas were selected randomly with the guidance from the Ramshah Path, Kathmandu, Metropolitan Traffic Police Division. The study sites are shown in Figure 1.

### 2.2. Sampling and Sample Size

Simple random sampling technique was used in the study. The list of all traffic police from each area was obtained from Metropolitan Traffic Police Division, Ramshah Path, Kathmandu. The sample was selected as whoever was available in the selected area to fulfill the criteria of the study. Therefore, a total of 83 samples were taken for the study. However, those traffic police who were involved in administrative work and had less than 6 months of work experience were excluded from the study.

### 2.3. Questionnaires and Data Collection Procedure

Close ended/open ended structured questionnaires were developed which included information regarding demography, knowledge, and practice on prevention of respiratory problems. All the questionnaires were translated into Nepali language for the ease of respondents (traffic police) to answer the questions. The content of the questionnaire was developed by an extensive consultation with the public health experts and Metropolitan Traffic Police Division, Ramshah Path, Kathmandu. The internal consistency of the questionnaires was obtained with Cronbach's alpha score of 0.83 for knowledge and 0.6 for practice.

Pretesting of the questionnaires was done with respondents at Kamalpokhari branch, Kathmandu. A total of nine traffic police officers were enrolled in the pilot study. After taking their feedback, seven out of the nine traffic police officers mentioned “use of antipollution mask” to protect from air pollution in knowledge-related questions. This was added in the final study. Similarly, information regarding “rotation in duty area” was added in the final study as five out of nine traffic police officers mentioned it. However, these pretest respondents were not included in actual study.

Self-administered questionnaires in Nepali language were distributed to all the study respondents. The respondents were briefed on the purpose of the study beforehand and they were briefed on the details of the questionnaires. Fifteen to thirty minutes was allotted for each subject for filling the questionnaires. The respondents filled the questionnaire in the presence of the researcher and the questionnaire was immediately collected.

### 2.4. Ethical Consideration

Ethical approval was taken from research committee of Nursing Campus, Maharajgunj. Similarly, permission was obtained from the Metropolitan Traffic Police Division, Ramshah Path, Kathmandu. Verbal and written informed consent was taken from each respondent before collecting data. Anonymity and confidentiality was maintained through the study and none was compelled to participate in the study.

### 2.5. Ranking of the Respondents

In the evaluation of level of knowledge of the respondents, 14 knowledge-related questions were included. Every right answer was awarded one mark and every wrong answer was awarded zero. In addition, multiple choice answer was awarded more than one mark for each correct answer. The total score of knowledge-related questions was 36. Similarly, in the evaluation of level of practice of the respondents, a total of five questions regarding the preventive practice for respiratory problems were included. The total score was five. The ranking of respondents was done as follows: below average (score < 50%), average (score of 50% to 69%), and above average (score ≥ 70%).

### 2.6. Data Processing and Management

Initially, the collected data was checked daily for its completeness. The data was entered to and analyzed in Statistical Package for Social Sciences (SPSS Inc., version 18). Descriptive statistics were made by using proportions, mean, and standard deviation (SD), while inferential calculation was made using Fischer's exact test. *P* value < 0.05 was considered significant.

## 3. Results

The mean age (±SD) of the respondents was 28.8 ± 4.3 years. Majority of the respondents (94.0%, *n* = 78) were male and nearly four-fifth of the respondents (79.0%, *n* = 66) were married. Regarding educational status, all the respondents had completed SLC (School Leaving Certificate) and almost half of them had passed intermediate level. More than half of the respondents had 6–10 years of work experience, the mean (±SD) years of experience being 7.9 (±3.6) (Table 1).

Among the effects of the pollution on respiratory system, the majority (84.3%, *n* = 70) responded with lung cancer followed by bronchial asthma (78.3%, *n* = 65). More than ninety percent (*n* = 75) responded with difficulty in breathing as a major sign and symptom when they get respiratory problems. Similarly, among the ways, nearly four-fifth reported that the use of mask and about three-fifth reported that regular health checkup can prevent the effects of air pollution. About three-fourth reported that antipollution mask was a suitable mask to prevent air pollution (Table 2).

**Figure 2:**
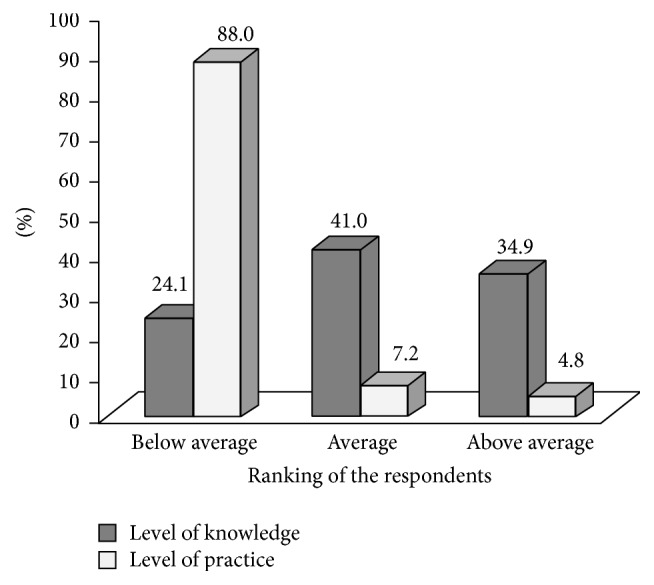
Level of knowledge and practice on prevention of respiratory problems.

88.0% (*n* = 73) of the respondents had below-average level of practice to prevent respiratory problems. Likewise, 41.0% (*n* = 34) of the respondents had average level of knowledge regarding the prevention of respiratory problems (Figure 2).

The majority of the respondents (94.0%, *n* = 78) used masks during duty hours while more than half of the respondents had rotation in duty areas in order to prevent respiratory problems. Less than one out of 10 respondents had regular health checkup (Table 3).

The level of practice was below average (I) in majority of respondents having intermediate level of education. In addition, there was no association between education and level of practice (Table 4).

The majority of respondents with 11 to 15 years of working experience had level of knowledge above average followed by respondents with 16 to 20 years of experience. 91.3% (*n* = 43) of respondents with experience of 6 to 10 years had level of practice below average. There was an association between working experience and level of practice (*P* = 0.04) (Table 5).

## 4. Discussion

The present study showed that the traffic police had knowledge regarding the negative effects of air pollution on their health. They had knowledge that air pollution can cause difficulty in breathing, wheezing sound, lung cancer, bronchial asthma, and pneumonia. Studies conducted in India have also found that there is increased risk of getting different respiratory problems when traffic police are exposed to polluted air for a longer time [2, 15]. It is essential for traffic police to be aware of the problems especially related to respiration in cities like Kathmandu. Although majority of the traffic police had knowledge that they need to use antipollution mask, fewer of them felt like going for regular health checkup. Regular checkups save lives even when there is no specific problem, since the absence of disagreeable symptoms does not necessarily guarantee that one is in good health. Some studies have also shown decreased rates of invasive cancers and decreased mortality in people who undergo regular medical checkup [16–18]. Therefore, awareness creating activities and policies relating to regular health checkup and protection from polluted environment should be launched effectively.

This study showed that the level of knowledge regarding the prevention of respiratory problems among the traffic police was comparatively higher than the level of practice. Similar results were obtained in a study done in India; the level of knowledge was found to be better in majority of traffic police while the level of practice was average [19]. This might be due to financial problems faced by the traffic police as studies have suggested that socioeconomic factors play a role in the health seeking behaviors. In addition, lack of time management during duty periods could prevent attending regular health checkup [20, 21]. Nonetheless, they should be motivated to have healthy and safe practice against the pollution. Furthermore, the government should draft sustainable policies addressing such issues.

The majority of traffic police used masks to prevent respiratory problems in this study. A study revealed that the use of facial masks could help to reduce the negative effects of air pollution [22]. A minority of the traffic police implemented rules in vehicles for less emission of gases in this study. This might be due to ineffective rules regarding prevention of vehicular emission. In Kathmandu Valley, vehicular emission is the major contributor to air pollution [9]. A study done in Kathmandu Valley showed that the total estimated emission (CO, CO_2_, HC, NO_X_, SO_2_, and PM_10_) was 7,231,053.12 tons/year, the majority of which was CO_2_ (91.0%) and CO (5.0%) [6]. In addition, at the time of this study, demolition of buildings and construction of roads were in progress. This could have aggravated the condition of air, eventually deteriorating the health of the traffic police. The good health of traffic police and city dwellers is ensured only if the government implements vehicle inspection and emission testing effectively and ban on polluting vehicles.

The present study showed that education affected the level of knowledge in prevention of respiratory problems among traffic police. However, there was no association between education and level of practice to prevent respiratory problems. The poor practice of the traffic police, despite better knowledge, has not been fully understood necessitating further research in this regard. This study also showed that working experience had association with the level of practice but not with the level of knowledge. A research work suggests that, with longer experience, people increase their level of performance [23]. It is better to learn preventive measures against air pollution from experience and practice them in daily life.

This study was not without limitations. First, all traffic stations in Kathmandu Metropolis could not be taken into account due to budget limitation. Second, it would have been better to observe the practices of the traffic police rather than to provide them with the questionnaires to fill them.

In the future, research can be done by providing some interventions like training to the traffic police and observing the outcome in their knowledge and practice. Other health effects such as hearing impairment, eye problems, hypertension, and respiratory functions can be studied among the traffic police.

## 5. Conclusion

More than ninety percent of the traffic police mentioned that difficulty in breathing was a major sign and symptom when they get respiratory problems. Among the ways, the use of mask and regular health checkup can prevent the effects of air pollution. The majority of the traffic police used masks during duty hours and had rotation in duty areas in order to prevent respiratory problems. The level of knowledge among the traffic police is adequate, but the level of practice on prevention of respiratory health problems is not satisfactory. Association of level of practice was observed with working experience but not with education of the traffic police in Kathmandu. It is the responsibility of the government and concerned authorities to deal with the issues and derive effective and sustainable solutions to ensure better health of traffic police in the city.

## Figures and Tables

**Table 1:** Biodemographic characteristics of the respondents (*n* = 83).

Variables	Frequency	Percent
Age (in years)		
20–24	10	12.0
25–29	40	48.2
30–34	21	25.3
35–40	12	14.5
Mean (±SD) = 28.8 ± 4.3		
Sex		
Male	78	94.0
Female	5	6.0
Marital status		
Married	66	79.5
Unmarried	16	19.3
Separated	1	1.2
Education		
SLC	28	33.7
Intermediate level	41	49.4
Bachelor's degree	10	12.0
Master's degree	4	4.8
Experience (in years)		
1–5	18	21.7
6–10	46	55.4
11–15	17	20.5
16–20	2	2.4

**Table 2:** Knowledge regarding respiratory problems due to air pollution and their prevention.

Variables	Frequency	Percent
Effects on respiratory system		
Pneumonia	36	43.4
Lung cancer	70	84.3
Bronchial asthma	65	78.3
Common cold	59	71.1
Sign and symptoms of respiratory problems		
Cough	48	57.8
Difficulty in breathing	75	90.4
Wheezing sound	59	71.1
Sneezing and running nose	44	53.0
Preventive measures to avoid respiratory problems		
Proper management of waste	55	66.3
Wearing mask	65	78.3
Regular health checkup	48	57.8
Less work on polluted area	49	59.0
Suitable mask to protect from air pollution		
Cloth mask	14	16.9
Antipollution mask	62	74.7
Gas mask	7	8.4

**Table 3:** Practice on prevention of respiratory problems.

Variables	Frequency	Percent
Use of masks	78	94.0
Decreased duty hours	10	12.0
Rotation in duty area	46	55.4
Regular checkup	8	9.6
Implement rules in vehicles regarding air pollution	5	6.0

**Table 4:** Association of education with level of practice on prevention of respiratory problems.

Education	Level of practice			
	I (%)	II (%)	III (%)	*P* value
SLC (*n* = 28)	25 (89.3)	2 (7.1)	1 (3.6)	0.32
Intermediate (*n* = 41)	37 (90.2)	2 (4.9)	2 (4.9)	
Bachelor's degree (*n* = 10)	8 (80.0)	2 (20.0)	0 (0.0)	
Master's degree (*n* = 4)	3 (75.0)	0 (0.0)	1 (25.0)	

*Note.* I: below average; II: average; III: above average.

**Table 5:** Association of experience with level of knowledge and practice on prevention of respiratory problems.

Experience (in years)	Level of knowledge				Level of practice			
	I (%)	II (%)	III (%)	*P* value	I (%)	II (%)	III (%)	*P* value
1–5 (*n* = 18)	6 (33.3)	7 (38.9)	5 (27.8)	0.33	15 (83.3)	1 (5.6)	2 (11.1)	0.04
6–10 (*n* = 46)	12 (26.1)	21 (45.6)	13 (28.3)		42 (91.3)	3 (6.5)	1 (2.2)	
11–15 (*n* = 17)	2 (11.8)	5 (29.4)	10 (58.8)		15 (88.2)	2 (11.8)	0 (0.0)	
16–20 (*n* = 2)	0 (0.0)	1 (50.0)	1 (50.0)		1 (50.0)	0 (0.0)	1 (50.0)	

*Note.* I: below average; II: average; III: above average.
